# Fermentative valorization of edible algae: nutritional potential, food applications, safety considerations, and industrial challenges

**DOI:** 10.3389/fmicb.2026.1966638

**Published:** 2026-10-26

**Authors:** Donglin Ma, Chaokang Xia, Aiming Jia, Guilan Zhang, Yuzong Zhao, Haiwei Ren, Xiaogang Liu, Fengjun Li, Lilang Li, Ruibo Jia, Kuntai Li

**Affiliations:** 1 Guangdong Provincial Key Laboratory of Aquatic Product Processing and Safety, College of Food Science and Technology, Guangdong Ocean University, Zhanjiang, Guangdong, China; 2 Tobacco Research Institute, Chinese Academy of Agricultural Sciences, Qingdao, China; 3 School of Life Science and Engineering, Lanzhou University of Technology, Lanzhou, Gansu, China; 4 Luzhou Laojiao Group Co., Ltd., Luzhou, Sichuan, China

**Keywords:** edible algae, fermentation, food safety, functional foods, precision processing, sustainable food systems

## Abstract

Edible algae, including microalgae and macroalgae, are recognized as sustainable aquatic bioresources for future food systems, with high areal productivity, low reliance on arable land and freshwater, and rich nutrients including proteins, polyunsaturated fatty acids, pigments, minerals, vitamins and functional polysaccharides. Yet their wider application in mainstream foods is limited by low digestibility, rigid cell walls, marine off-flavors, variable safety and high processing costs. This review examines the nutritional potential, food applications, fermentation strategies, safety considerations and industrial bottlenecks of edible algae, focusing on fermentation-mediated valorization as an enabling strategy beyond conventional preservation. Lactic acid, alcoholic and mixed-culture fermentation can boost shelf stability, release intracellular nutrients, modulate sensory attributes, generate bioactive metabolites, and support circular use of residual algal biomass. Meanwhile, advanced technologies like enzyme-assisted extraction, non-thermal extraction, microfluidics and nanoencapsulation help enhance ingredient functionality and bioactive delivery. The review also notes that the future value of algae-based foods relies on matrix-specific formulation, standardized safety control for heavy metals and iodine, strain improvement, biorefinery integration and consumer-oriented sensory design. Overall, fermentative and precision-processing strategies offer a promising route to transform algae from niche supplements into safe, palatable, multifunctional ingredients for sustainable food innovation.

## 1 Introduction

One of the foremost challenges of the 21st century is ensuring adequate food supply for a growing global population amid increasingly constrained natural resources. By 2050, the world population is expected to approach 9 billion, creating a strong demand for sustainable sources of protein, lipids, micronutrients, and functional food ingredients (Vos and Bellù, 2019). In this context, edible algae including both microalgae and macroalgae, have attracted considerable attention because they can be cultivated in aquatic or controlled systems, generally require less arable land than terrestrial crops, and exhibit high biomass productivity. Compared with conventional terrestrial crops, many algal species can be cultivated with limited reliance on arable land and freshwater, while exhibiting high areal productivity and the capacity to grow in marine, brackish, or otherwise non-arable environments (Wu et al., 2025). These features make algae particularly relevant to the development of diversified food systems that are less dependent on conventional agricultural inputs.

The use of algae as food is not new. Archaeological evidence indicates that algae were consumed approximately 14,000 years ago at the Monte Verde site in Chile, and seaweeds remain important dietary components in many coastal cultures (Mendes et al., 2022). Taxonomically, algae encompass a broad range of photosynthetic organisms, including Phaeophyceae, Chlorophyta, Cyanophyta, and Rhodophyta, with substantial variation in morphology, habitat, composition, and technological functionality. Several microalgal species have already entered commercial food and nutraceutical markets. The U.S. Food and Drug Administration has granted Generally Recognized as Safe (GRAS) status to selected species, including Arthrospira platensis, *Chlamydomonas reinhardtii*, *Auxenochlorella protothecoides*, *Chlorella vulgaris*, *Dunaliella bardawil*, and *Euglena gracilis* (Torres-Tiji et al., 2020). Among them, Arthrospira platensis and *Chlorella vulgaris* are among the most widely commercialized edible microalgae and are also recognized for food use in Europe (Chacón-Lee and González-Mariño, 2010).

The nutritional value of edible algae underpins their growing relevance in food innovation. Chlorella, for example, has attracted substantial commercial interest because its dry biomass may contain 51–58% protein, together with essential amino acids, β-1,3-glucan, B-complex vitamins, ascorbic acid, minerals, β-carotene, chlorophyll, and Chlorella growth factor (Silva et al., 2019). Dried biomass of Arthrospira and Chlorella currently represents the largest share of global microalgal production, with estimated annual production of approximately 5,000 tonnes of dry matter for Arthrospira and 2,000 tonnes for Chlorella, corresponding to a combined market value of about US$40 million per year (Kraemer, 2021). Beyond whole biomass, edible algae provide proteins, polyunsaturated fatty acids, vitamins, minerals, pigments, and functional polysaccharides, making them promising ingredients for food fortification, texture modification, active packaging, and functional food formulation. Algae-derived biopolymers and biochar-based materials have also shown potential in sustainable packaging and analytical applications, reflecting the broader relevance of algal resources in food-related technologies (Pruteanu et al., 2024; Yue et al., 2024). However, the translation of algal biomass into mainstream food products remains constrained by several unresolved issues. These include rigid cell-wall structures, limited digestibility, marine off-flavors, green coloration, variable composition, safety concerns related to heavy metals and iodine, and technically demanding downstream processing (Dewan et al., 2025). These limitations indicate that simple biomass addition is insufficient for successful product development.

Fermentation provides a promising strategy to address some of these barriers. Compared with conventional physical or chemical processing, microbial fermentation can contribute to cell-wall disruption, nutrient release, flavor modulation, shelf-life extension, and the generation of value-added metabolites. More importantly, fermentation can support the conversion of algal biomass and residual streams into multifunctional food ingredients within a biorefinery-oriented framework. Nevertheless, the effectiveness of fermentation depends strongly on algal species, substrate composition, microbial strains, processing conditions, and target food matrices. Therefore, a more systematic understanding is required to connect algal composition, fermentation mechanisms, product functionality, safety control, and industrial feasibility.

This review critically examines the nutritional potential, food applications, fermentation strategies, safety considerations, and industrial challenges associated with edible algae. Particular emphasis is placed on fermentation-mediated valorization as an enabling strategy for transforming algae from niche supplements into safe, palatable, and multifunctional food ingredients. By integrating evidence on algal composition, component–function–matrix matching, precision processing, safety governance, and biorefinery-oriented utilization, this review aims to provide a coherent framework for advancing algae-based foods within sustainable and innovation-driven food systems (Figure 1).

**Figure 1:**
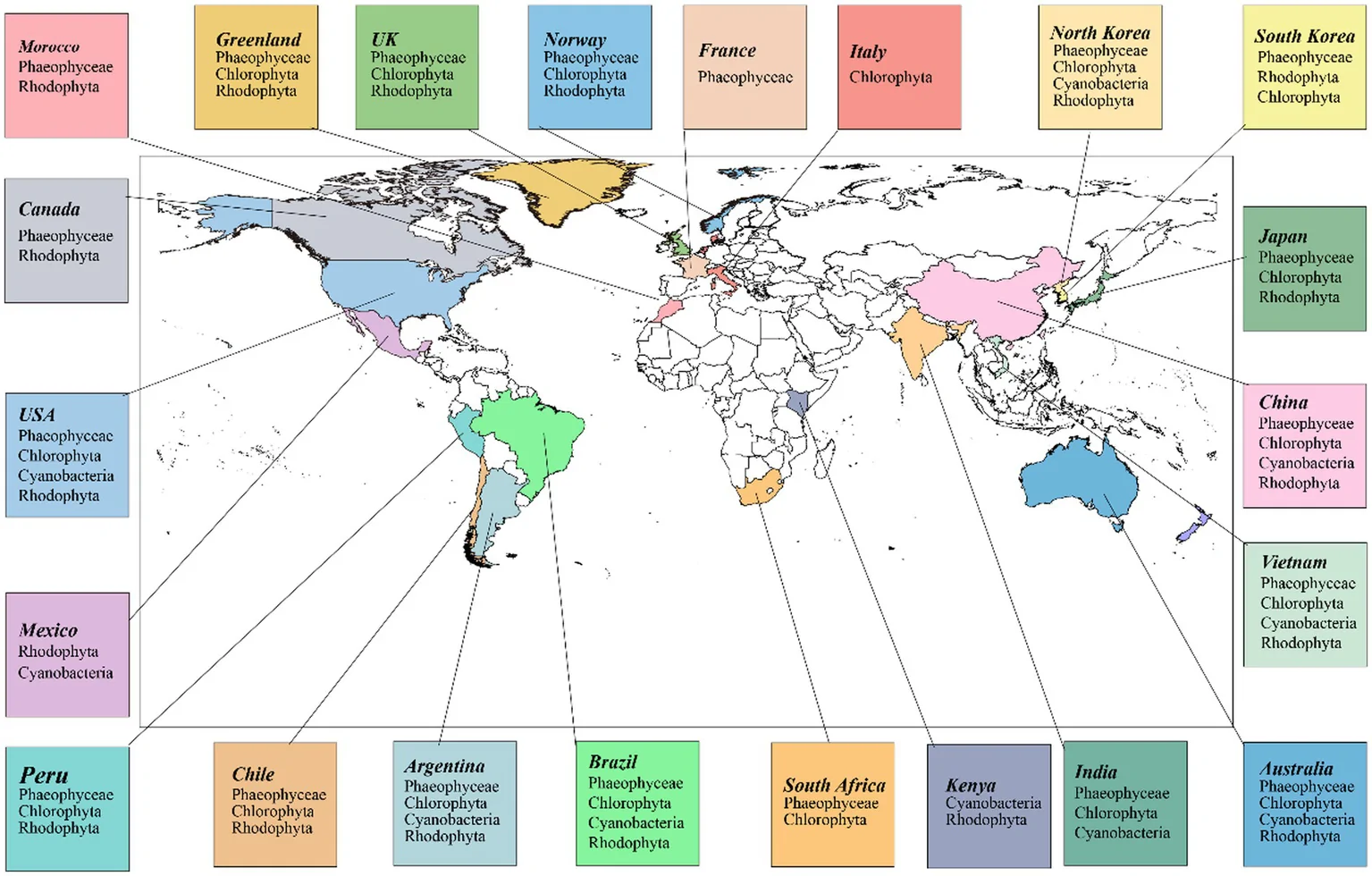
The growth and distribution of different algae across global countries.

## 2 Nutritional composition and functional value of edible algae

### 2.1 Protein and amino acids

Algae and microalgae have been recognized as feasible sources of dietary protein. Certain species display protein concentrations comparable to those of traditional sources like meat, eggs, soy, and milk. For instance, the red algae *Palmaria palmata* and *Porphyra purpurea, respectively,* exhibit protein contents reaching up to 20 and 30% of dry weight (Echave et al., 2021). Likewise, a multitude of microalgae species manifest significantly higher protein contents. *Arthrospira*, for example, has been documented to contain up to 60% protein dry weight (Ragaza et al., 2020). It is among the most extensively consumed microalgae, esteemed not merely for its high protein concentration but also for its proven health - promoting properties, encompassing antihypertensive, renoprotective, antihyperlipidemic, and antihyperglycemic activities. Beyond its protein fraction, Arthrospira is an abundant source of gamma - linolenic acid, which contributes to cholesterol reduction, B vitamins, and phycobiliproteins that possess free radical–scavenging capacity (ElFar et al., 2022). These acknowledged nutritional attributes have prompted the World Health Organization to label it as a superfood and its utility has been evidenced even in NASA space missions (Shaw and Soma, 2022). Nonetheless, plant - based proteins, including those sourced from algae, are often classified as incomplete owing to typical deficiencies in one or more essential amino acids. In most algal species, lysine and tryptophan are the principal limiting amino acids (Bleakley and Hayes, 2017). Moreover, the amino acid profiles of numerous algae are characterized by a high prevalence of aspartic and glutamic acids, which significantly contributes to the distinctive umami taste of algal - based products. From the perspectives of productivity and nutrition, algae offer considerable advantages over conventional high - protein crops such as soybeans. While terrestrial crops like soybeans, pulses, and wheat yield approximately 0.6–1.2, 1–2, and 1.1 tons of protein per hectare per year respectively, microalgae and macroalgae can attain substantially higher protein yields, reaching 4–15 and 2.5–7.5 tons per hectare per year respectively (Mounaimi et al., 2026).

### 2.2 Lipids and polyunsaturated fatty acids

Lipids serve as indispensable cellular constituents and precursors of numerous essential biomolecules, rendering their adequate intake of critical significance for human health. Certain algal species possess the capacity to accumulate lipids to remarkably elevated levels. For example, *C. vesiculifera f. auxiliaris* can store lipids accounting for up to 70% of its dry biomass (Satpati and Pal, 2021). In comparison with terrestrial plants and animals, algae are conspicuously rich in health - promoting compounds, encompassing dietary fiber, ω-3 polyunsaturated fatty acids, essential amino acids, and vitamins A, B, C, and E. Humans generally obtain these nutrients through the consumption of seafood, as numerous marine organisms subsist on planktonic algae. Macroalgae, in particular, accumulate a diverse spectrum of nutrients, such as proteins, minerals, vitamins, dietary fiber, fatty acids, pigments, and other bioactive compounds. These organisms synthesize essential long - chain PUFAs that are requisite for human physiology (Tagliapietra and Clerici, 2023). Specific microalgae, including the diatom *Phaeodactylum tricornutum*, can accumulate eicosapentaenoic acid to levels constituting 30–40% of their total fatty acid profile. Other species, such as *Schizochytrium limacinum*, can allocate nearly 50% of their total lipid content to docosahexaenoic acid (Wang Q. et al., 2021). Therefore, algae represent a viable and sustainable alternative to traditional fish oil supplements, providing a direct, contaminant-free source of these essential, health - promoting fatty acids.

### 2.3 Vitamins, minerals, pigments, and other bioactives

Edible algae can supply vitamins, minerals, carotenoids, chlorophylls, phycobiliproteins, phenolics, sulfated polysaccharides, and other bioactive compounds. Algae and microalgae serve as particularly abundant sources of bioavailable nutrients. For instance, the green algae *A. chlorophyla* has been recognized as a significant provider of vitamins A, B1, B9, and E, indicating that algae biomass or extracts can directly furnish nutrients crucial for human development (Del Mondo et al., 2020).

In addition to vitamins, algae offer other health - promoting compounds, including antioxidants like lycopene, β-carotene, and astaxanthin, as well as bioactive polysaccharides such as β-glucan. Moreover, it has been demonstrated that the dietary consumption of certain microalgae can support immune modulation and contribute to a wide range of health benefits. These benefits encompass improved cardiovascular function, enhanced immune response, anti-aging effects, and anticancer properties (Figure 2) (Fields et al., 2020).

**Figure 2:**
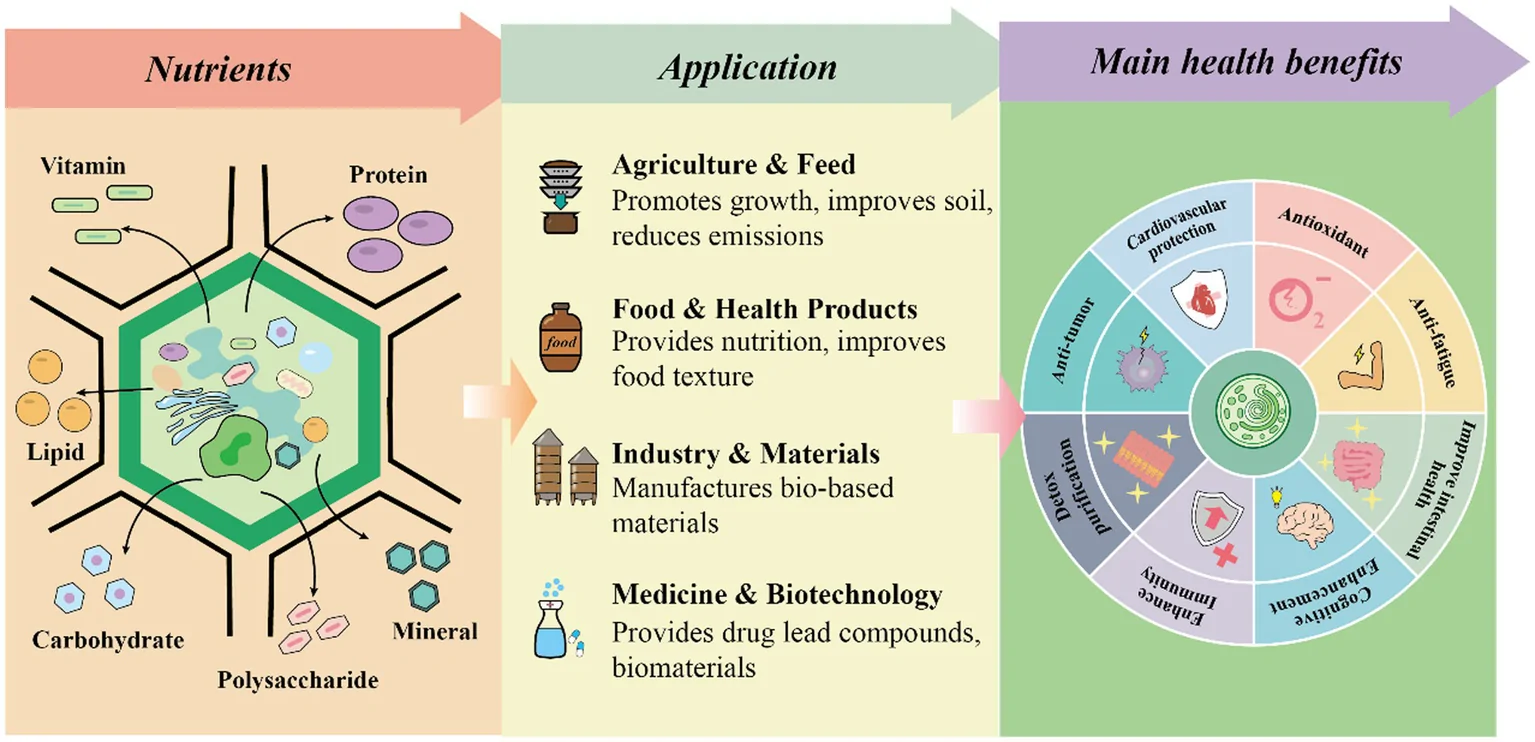
Nutritional composition and function of algae.

### 2.4 Functional polysaccharides

Algal polysaccharides, including agar, alginate, and carrageenan, are principal structural constituents of the cell walls of brown and red macroalgae and have achieved extensive industrial application. Macroalgae stand as the primary source of agar, with specific species demonstrating remarkably high agar content. For example, the red algae *G. gracilis* contains 30 and 27% agar in specimens originating from different geographical regions (Sfriso et al., 2017). In the food industry, agar is extensively utilized as a thickening and gelling agent due to its thermoreversible gelation behavior and the capacity to form stable gels at low concentrations. The high agar yield and cost-effectiveness of macroalgal biomass render it a preferred raw material for industrial agar production, substantially facilitating the development of food-processing technologies.

Alginate, an acidic polysaccharide predominantly extracted from brown algae such as *Sargassum* spp., can account for up to 40% of the dry weight in certain species, highlighting its abundance in marine biomass. This polymer plays a crucial role in food processing and packaging due to its gelling, thickening, and stabilizing functions. Alginate hydrogel particles are among the most widely employed encapsulation systems, recognized for their biocompatibility, non-toxicity, biodegradability, low cost, and ease of production. Research by Steva Lević et al. indicated that compounds encapsulated in Ca-alginate gel particles do not have an adverse impact on flavor release during consumption (Lević et al., 2015). Beyond food applications, alginate holds significant value in the biomedical field. As a key structural component of brown algal cell walls, alginate typically constitutes 10–40% of algal dry mass (Yarkent et al., 2022). For instance, the alginate content in Ghanaian brown algae of the genera *Sargassum* and *Padina* ranges between 16 and 30%, while *Sargassum wightii* from Sri Lanka yields sodium alginate at 31.7% (Jayasinghe et al., 2022; Rhein-Knudsen et al., 2017). Therefore, macroalgae represent a sustainable and abundant source of alginate with far - reaching implications for food and medical applications.

Carrageenan, a class of sulfated polysaccharides primarily derived from red macroalgae, exhibits gelling, thickening, and stabilizing properties, making it a valuable additive in the food industry as a thickener and stabilizer. Its content in algal biomass generally ranges from 15 to 50%, with the main commercial types being kappa (κ), iota (ι), and lambda (λ) carrageenans (Tarman et al., 2020). These variants display distinct functional characteristics due to differences in their chemical structures. For example, κ - carrageenan, which has a higher 3,6 - anhydrogalactose content and fewer sulfate ester groups, forms strong and brittle gels, particularly in the presence of K^+^ (Gomez-Zavaglia et al., 2019). In contrast, t-carrageenan, which has a higher degree of sulfation, produces soft and elastic gels, especially in the presence of calcium ions (Gomez-Zavaglia et al., 2019). As a versatile algal polysaccharide, carrageenan has promising applications in the food, pharmaceutical, and cosmetic sectors. Moreover, ongoing advancements in extraction technology are anticipated to further enhance carrageenan production, utilization efficiency, and value.

### 2.5 Biological characteristics, habitat, and distribution

The nutritional and technological value of algae is inseparable from their biological diversity. Algae include Rhodophyta, Chlorophyta, Phaeophyceae, and Cyanobacteria and occur in marine, freshwater, and terrestrial habitats. Their ecological functions include carbon fixation, oxygen release, nutrient cycling, metal-ion adsorption, and mitigation of eutrophication. Most macroalgae grow optimally at 15–25 °C and moderate salinity, although some species exhibit strong tolerance to extreme environments. For example, *D. salina* can grow under alkaline conditions up to pH 11.0, whereas the optimal pH for many macroalgae is around 7.4 (Scherner et al., 2016; van Ginneken, 2018). This ecological plasticity explains the wide distribution and diverse composition of edible algae. Freshwater taxa such as Nostoc, Oedogonium, Chara, and Cladophora commonly occur in rivers, ponds, and lakes, while many macroalgae dominate intertidal zones, rocky shores, coral reefs, and seagrass habitats. For instance, *P. stipitata* colonizes intertidal rocks and obtains nutrients through mineral weathering (Brazier et al., 2019). Coastal surveys have reported high algal diversity, including 69 species in seagrass beds along the Fudao coastline and 13–18 macroalgal species at Derini and Kongdang Merak beaches (Hieu et al., 2019; Romdoni et al., 2018). Korean natural algal beds also support communities with 5–48 species on average (Jung et al., 2022). Such diversity provides a broad resource base for food development, but it also means that composition, safety, and processing behavior vary substantially among species and regions (Table 1).

**Table 1:** Habitats and applications of different algae.

Representative species	Growing regions	Core advantage nutrients	Application	References
*Laminaria japonica*	coastal areas in cold waters, the North Atlantic, and the Pacific Ocean	Polyphenols, fucoidan, iodine, proteins, and carbohydrates	Soup bases and stews	Jayathilake and Costello (2020)
*Macrocystis pyrifera*	Temperate and subpolar coastlines	Fucoidan, alginate, and dietary fiber	Feed	Biparva et al. (2023) and Shen et al. (2021)
*Undaria pinnatifida*	Temperate regions, the northwest Pacific Ocean	Polyphenols, sulfated fucoidan, proteins, carbohydrates, vitamins A, C, E, calcium, and magnesium	Salads and soups	De Leij et al. (2017) and Shen et al. (2021)
*Sargassum spp*	Tropical and temperate coasts	Proteins carbohydrates, PUFAs, dietary fiber sodium, potassium, calcium, magnesium, iron, zinc, copper, flavonoids, and polyphenols	TCM and health products	Li and Du (2024) and Premarathna et al. (2022)
*Sargassum fusiforme*	Temperate coastal areas and intertidal zones in East Asia	Phenolic compounds	Antioxidant	Shen et al. (2021) and Xiao-mei et al. (2018)
*Sargassum thunbergii*	Temperate and tropical forests	Polysaccharides, dietary fiber, and phenolic compounds	Soup bases and feed	González-Gallegos et al. (2020)
*Sargassum pallidum*	Temperate coastal regions	Polysaccharides and minerals, phenolic compounds	Immune enhancer	Zhang et al. (2021)
*Scytosiphon lomentaria*	Temperate coastal regions	Polysaccharides and phenolic compounds	Feed	Croce et al. (2023)
*Ishige okamurae*	Intertidal and subtidal zones	Kelp polyphenols	Salad and soup base	Lee (2015)
*Codium fragile*	Temperate and subtropical marine areas	Crude polysaccharides, carbohydrates, proteins, and phenolic compounds	Food additives	Ortiz et al. (2009)
*Ulva prolifera*	Inland lake waters, intertidal zones, planktonic substrates	Proteins, dietary fiber, vitamins, and minerals	Feed	Cho et al. (2010) and Ding et al. (2009)
*Monostroma nitidum*	On intertidal rocks in marine and estuarine habitats	Phenolic compounds	Salads and soups	Kaur et al. (2023) and Peñalver et al. (2020)
*Ulva lactuca*	Freshwater lakes, coastal rocks (for euryecological species)	Proteins, carbohydrates, dietary fiber, lipids, and phenolic compounds	Dietary fiber supplement	Dominguez and Loret (2019) and Premarathna et al. (2022)
*Porphyra*	High-tide zones in temperate and subtropical marine areas	Proteins, B vitamins, vitamins A, C, iron, calcium, potassium, and crude polysaccharides	Crispy Snacks	Cian et al. (2014)
*Gelidium*	High-tide and low-tide zones along the Pacific coast	Dietary fiber and phenolic compounds	Jellies, Puddings, and Bread	Boo et al. (2014) and Siddique et al. (2013)
*Gloiopeltis furcata*	coasts of the southwestern Atlantic	Dietary fiber, proteins, minerals, phycobiliproteins, and phenolic compounds	Agar	Cebrian-Lloret et al. (2024) and Warnasooriya et al. (2024)
*Nostoc flagelliforme*	In deserts, arid soils, and on rock walls across all continents	Sulfated polysaccharides	Dietary Supplements	Aboal et al. (2016)
*Spirulina*	Subtropical to tropical oceans	Proteins, B vitamins, β-carotene, vitamin E, calcium, iron, magnesium, zinc, selenium, phycocyanin, β-carotene, and γ-linolenic acid	Thickener	Ramírez-Rodrigues et al. (2021)
*Leathesia difformis*	Temperate coastal areas of the Atlantic, attached to rocks and other Algae	Fucoidan, alginate, iodine, and dietary fiber		Jeong-Ryong et al. (1997)

### 2.6 Reproductive traits and implications for strain improvement

Algal reproductive systems are diverse and directly relevant to domestication and strain improvement. Many algae alternate between haploid and diploid phases. Diploid-dominant species produce haploid gametes that fuse into diploid zygotes, whereas haploid-dominant species undergo meiosis soon after zygote formation (Nelson and Zuccarello, 2025). Haplodiplontic life cycles contain mitotic divisions in both haploid and diploid stages, and the two generations may be morphologically similar or heteromorphic. In addition, both haploid and diploid stages may reproduce asexually through vegetative fragmentation, asexual spores, or parthenogenesis (Liu et al., 2017). Red algae often possess an additional carposporophyte stage that remains nutritionally dependent on the female gametophyte (Mouga and Fernandes, 2022).

This reproductive plasticity enhances ecological adaptation but complicates breeding. Unlike many terrestrial crops, most edible algae have not undergone systematic domestication. Therefore, clarifying life cycles, ploidy regulation, and reproductive triggers will be essential for developing high-yielding strains with improved nutrient profiles, reduced off-flavors, better digestibility, and stable performance under industrial cultivation conditions (Liu et al., 2017).

## 3 Utilization and fermentation of edible algae for food production

### 3.1 Food applications: from nutrients to structured ingredients

Algae can be incorporated into food systems as whole biomass, nutrient-rich powders, hydrocolloids, extracts, fermented substrates, or active packaging components. Their application value lies not only in nutrient density but also in their ability to provide texture, color, flavor precursors, antioxidant capacity, and bioactive functionality. Algal polysaccharides can form three-dimensional gel networks through intermolecular interactions, thereby converting liquid systems into semi-solid gels. In parallel, secondary metabolites such as polyphenols, carotenoids, sterols, lectins, halogenated compounds, polyterpenoids, and alkaloids contribute to antioxidant and disease-preventive potential (Babich et al., 2022).

A more coherent strategy is to match algal components with suitable food matrices. Protein-rich microalgae are suitable for nutritional fortification, polysaccharide-rich macroalgae are suitable for texture modification and packaging, and bioactive-rich extracts are more appropriate for functional foods with targeted claims. This component–function–matrix matching represents a more innovative and practical route than simply adding algal biomass to foods.

### 3.2 Nutritional supplements and food fortification

Algae have been widely used as supplements, food additives, fortifying ingredients, and carriers for novel food formulations. Spirulina and *Chlorella vulgaris* may contain 50–70% protein on a dry-weight basis, making them attractive for protein enrichment. During peak growth, *Padina pavonica* can accumulate 12.8% protein, 14.3% lipids, and considerable dietary fiber, supporting its use as a nutrient-dense ingredient (Vasileva and Ivanova, 2019). Algal powders have also been incorporated into bakery, pasta, and dairy products. For example, mixed powders containing Spirulina and *Himanthalia elongata* improved the protein and dietary-fiber content of whole-wheat pasta while maintaining cooking stability and antioxidant capacity.

However, fortification must be formulation-specific. Inclusion levels should be optimized according to sensory compatibility, nutrient bioavailability, safety, color stability, odor, and texture. Excessive addition may reduce consumer acceptance, particularly in products with mild flavor or pale color. Thus, algal fortification should be guided by product-specific formulation design rather than by nutrient enrichment alone.

### 3.3 Hydrocolloid additives and edible packaging

Algae are rich in water-soluble hydrocolloids, including carrageenan, agar, and alginate, which are mainly derived from Rhodophyta and Phaeophyta. These compounds act as effective thickeners, gelling agents, emulsifiers, and stabilizers in food systems. Algal hydrocolloids improve gel stability, texture, and appearance in food products. They can also be extracted and incorporated into film-forming solutions to create active packaging materials (Carpintero et al., 2023). Alternatively, integrating algal extracts or biomass into protein- or polysaccharide-based matrices can enhance the mechanical strength and barrier properties of edible films. These films have shown significant potential for coating and preserving meat, fish, and fresh produce. For instance, sodium alginate, due to its excellent film-forming ability and ionic cross-linking capacity, has been widely used to prepare edible packaging films that significantly prolong the shelf life of fruits and vegetables. Therefore, water-soluble algae extracts serve as functional food additives and valuable components in the development of preservation-oriented food packaging.

### 3.4 Whole-algae ingredients and downstream processing routes

Whole algae can be directly used as food ingredients because they provide distinctive textures, marine flavors, and visible natural identity. Macroalgae, such as *Saccharina japonica*, *Pyropia* spp., and *Undaria pinnatifida*, have been conventionally incorporated into soups, salads, and sushi in various cultures worldwide. Moreover, the bioactive compounds in algae, including terpenoids, sterols, and glycolipids, present considerable potential for their use as primary components in products like seaweed bars and cheese–based snacks (Stright et al., 2025).

Nevertheless, the direct use of whole algae is limited by marine odor, strong color, tough cell walls, and variable digestibility. Enzyme-assisted extraction can improve the recovery of proteins and other intracellular compounds from algal biomass and thereby support industrial processing (Naseri et al., 2020). Fermentation may further improve flavor, nutrient release, and shelf stability, but its mechanisms and product innovations should be discussed in a dedicated fermentation section to avoid repetition.

## 4 Algae-based food products and precision processing strategies

Algae-based foods are moving beyond their traditional role as dietary supplements toward more sophisticated food systems that integrate nutrition, functionality, sustainability, and process innovation. Compared with conventional plant ingredients, algae provide a distinctive combination of proteins, polyunsaturated fatty acids, pigments, polysaccharides, minerals, and secondary metabolites. The value of algae in food applications therefore lies not only in nutrient enrichment, but also in their capacity to act as functional, structural, and biologically active components in next-generation food matrices. To avoid overlap with the preceding sections on basic composition, this section focuses on product-oriented applications and fermentation-enabled upgrading strategies.

### 4.1 Functional and health-oriented product design

The development of algae-based functional foods is mainly driven by the presence of bioactive compounds with antioxidant, antimicrobial, antiviral, antitumor, antihypertensive, neuroprotective, and immunomodulatory activities (Dolganyuk et al., 2020). These properties support the use of algae as sustainable ingredients for health-oriented food formulation. However, from a reviewer’s perspective, it is important to distinguish between demonstrated nutritional functionality and overextended therapeutic claims. Current evidence more strongly supports the role of algae in dietary risk reduction and metabolic support rather than direct disease treatment.

Microalgae and macroalgae have been increasingly incorporated into foods such as beverages, pasta, ready-to-eat bars, dairy products, fermented products, confectionery, and meat analogues. Species including *Euglena gracilis*, Spirulina, and Chlorella are especially suitable for whole-cell or powdered applications because of their high nutrient density and commercial availability (Mendes et al., 2022). In parallel, selected microalgae provide commercially valuable carotenoids, including β-carotene, astaxanthin, fucoxanthin, and lutein, which can improve both the nutritional and functional profiles of food products (Mendes et al., 2022). Future products should therefore be developed through matrix-specific design, in which species selection, active components, processing conditions, and sensory properties are jointly optimized.

### 4.2 Sustainable novel foods and advanced processing

Algae are increasingly regarded as strategic resources for sustainable novel foods because their cultivation requires limited arable land and freshwater while contributing to carbon fixation. Nevertheless, the sustainability advantage of algae can only be fully realized when cultivation, harvesting, extraction, formulation, and residual biomass valorization are considered as an integrated chain. A key innovation in recent studies is the shift from simple biomass addition to precision delivery and stabilization of algae bioactives.

Advanced technologies such as microfluidics, nanoencapsulation, ultrasound-assisted extraction, and microwave-assisted extraction provide new opportunities to improve the functionality and processability of algal ingredients. For example, microfluidic and nanoencapsulation systems can enhance the gastrointestinal delivery, stability, and bioavailability of algal protein hydrolysates (Mohammadi et al., 2023). Non-thermal extraction technologies can improve the recovery of bioactive compounds while reducing thermal degradation and solvent consumption (Marques et al., 2024). These approaches are particularly relevant because many algal compounds are sensitive to heat, oxidation, pH variation, and matrix interactions.

From an innovation perspective, algae-based novel foods should not be positioned merely as substitutes for animal- or plant-derived ingredients. Rather, they should be developed as multifunctional food platforms that combine sustainable biomass production, targeted nutrition, structural functionality, and controlled bioactive delivery. This positioning better reflects the unique contribution of algae to future food systems and aligns with the development of personalized nutrition and Food Industry 4.0.

## 5 Fermentation as a strategy for algal valorization

Algae have emerged as a highly promising substrate in the fermentation industry because of their distinctive biological characteristics, rich nutritional composition, and sustainable production potential. For example, Brown algae and microalgae are particularly abundant in carbohydrates, proteins, lipids, vitamins, and minerals, which can serve as direct or indirect substrates for microbial fermentation. Furthermore, algae exhibit exceptionally high photosynthetic efficiency and biomass accumulation rate. Certain species can achieve daily biomass growth rates exceeding 30% under optimal conditions, significantly outperforming traditional terrestrial crops. This rapid growth ensures both stability and scalability of the biomass supply, thereby establishing a viable foundation for industrial-scale fermentation processes (Figure 3).

**Figure 3:**
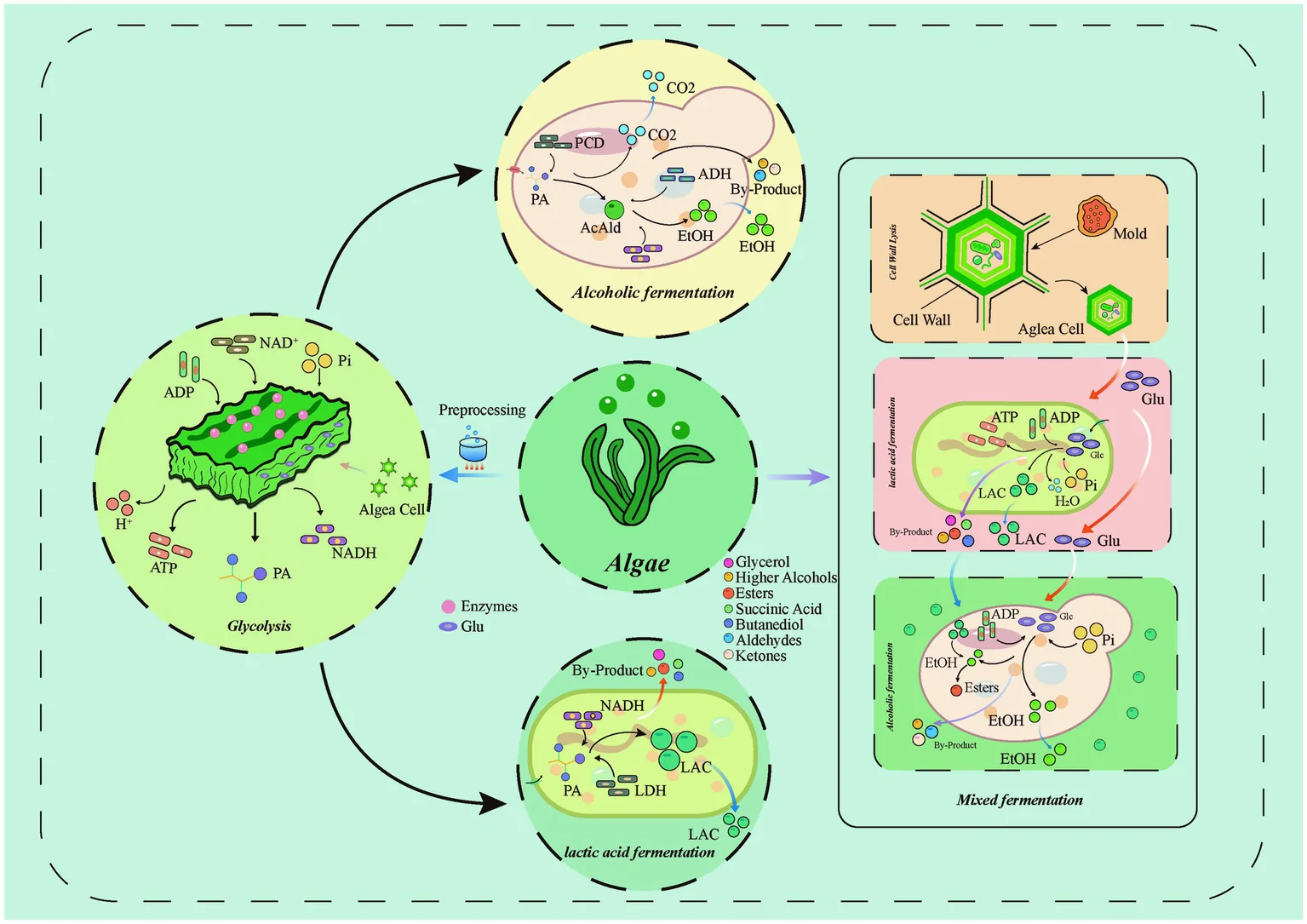
Mechanisms and Products of lactic acid fermentation, alcoholic fermentation, and mixed fermentation of aglae.

Microbial fermentation offers a transformative alternative to conventional physical and chemical extraction methods, which are often hindered by high energy consumption, significant degradation of bioactive compounds, and generation of high-salinity wastewater. This approach introduces three primary innovations in algal processing. First, fermentation using specific microbial strains facilitates the decomposition of algal cell walls, thereby releasing otherwise inaccessible nutrients, enhancing bioavailability, and reducing undesirable off-flavors. Second, it supports the development of novel food matrices, such as algal-based fermented products and mycoprotein derived from algae, thereby opening new market segments, including functional beverages and plant-based meat analogs. Third, mixed-culture fermentation enables the valorization of residual algal biomass and high-salinity wastewater, achieving near-zero waste processing and generating additional environmental value. In the context of increasingly stringent environmental regulations and emerging carbon trading mechanisms, this integrated strategy provides a competitive advantage to industrial stakeholders.

### 5.1 Mechanistic basis of fermentative valorization of edible algae

Fermentative valorization begins with the partial disruption of algal cell walls and the conversion of insoluble macromolecules into soluble compounds. Cellulases hydrolyze cellulose into oligosaccharides and glucose, whereas alginate lyases depolymerize alginate into alginate oligosaccharides. The combined use of alginate lyase and cellulase improved the hydrolysis of *Laminaria japonica*, indicating that enzyme combinations may be more effective than individual enzymes for deconstructing brown-algal biomass (Sun et al., 2020). In red algae, agarase and carrageenase producing bacteria can degrade agar and carrageenan, although these activities are strongly strain-dependent (Kang and Kim, 2015). Similarly, the degradation of the *Ulva lactuca* cell wall requires specific carbohydrate-active enzymes, including ulvan lyases and glycoside hydrolases (Costa et al., 2022).

During fermentation, microorganisms produce extracellular proteases, peptidases, glycosidases, and other carbohydrate-active enzymes. Proteolysis converts algal proteins into peptides and free amino acids, potentially improving protein digestibility, nitrogen availability, and flavor development. Mixed fermentation of *Porphyra yezoensis* with *Lactiplantibacillus plantarum*, *Bacillus amyloliquefaciens*, and *Saccharomyces cerevisiae* promoted the formation of antioxidant peptides, supporting the contribution of microbial proteolysis to the generation of functional compounds (Yang et al., 2024). *Filamentous-fungal* solid-state fermentation may further enhance enzyme production and modify the nutritional composition of algal biomass (Ferreira et al., 2022). The released sugars and amino acids subsequently enter microbial metabolic pathways.

Fermentation performance is determined by the interaction between algal composition, cell-wall structure, and microbial metabolism. For example, the rigid cell wall and high protein, pigment, and lipid contents of the freshwater microalga *Chlorella vulgaris* may limit nutrient accessibility and contribute to undesirable odors, while compositional differences can affect LAB growth and volatile-compound formation (Nicolotti et al., 2025). The brown macroalga *Alaria esculenta* contains alginate, mannitol, and laminarin-derived oligosaccharides; a LAB consortium utilized mannitol and laminari-oligosaccharides but showed limited utilization of mannuronic and guluronic acids (Allahgholi et al., 2023). The red macroalga Porphyra contains proteins and sulfated polysaccharides, and LAB fermentation has been reported to promote acidification, proteolysis, flavor modification, and changes in antioxidant capacity (Yang et al., 2022). The green macroalga *Ulva lactuca* contains cellulose and ulvan, whose cross-linked structure can restrict nutrient accessibility; enzyme-assisted pretreatment with ulvan lyase and other carbohydrate-active enzymes may therefore improve its fermentability (Costa et al., 2022). These examples demonstrate that microorganism selection and fermentation conditions should be tailored to the biochemical composition and structural characteristics of each algal species. These substrate–microorganism relationships are summarized in Table 2.

**Table 2:** Relationship between algal characteristics and fermentation outcomes.

Algal group and representative species	Major biochemical characteristics	Main structural or technological barriers	Suitable microorganisms and processing approaches	Reported fermentation outcomes	References
Freshwater microalgae, e.g., *Chlorella vulgaris*	Relatively high protein, lipids, pigments, vitamins, and intracellular carbohydrates	Rigid cell wall, limited nutrient accessibility, green or sulfur-like off-flavors	LAB fermentation; mechanical or enzymatic cell disruption; aroma-oriented fermentation	Acidification, modification of volatile compounds, and potential improvement of aroma quality	Nicolotti et al. (2025)
Edible cyanobacteria and microalgae, e.g., *Arthrospira*, *Euglena*, *Nostoc*, and *Dunaliella*	Proteins, pigments, polyunsaturated lipids, vitamins, and bioactive compounds	Species-dependent digestibility, pigment-related color, and possible off-flavors	LAB or yeast fermentation; species-specific starter selection	Changes in antioxidant and antiglycation activities; modification of sensory properties	Kaga et al. (2021)
Brown macroalgae, e.g., *Saccharina*, *Laminaria*, and *Alaria*	Alginate, laminarin, mannitol, fucoidan, minerals, and phenolic compounds	Complex alginate-rich cell wall; limited utilization of mannuronic and guluronic acids	LAB consortia, adapted yeasts, alginate lyase/cellulase pretreatment, or mixed cultures	Lactic acid formation, production of organic acids and alcohols, polysaccharide depolymerization, and flavor modification	Healy et al. (2023)
Red macroalgae, e.g., *Porphyra*, *Pyropia*, and *Gracilaria*	Agar, carrageenan, sulfated galactans, proteins, minerals, and pigments	Sulfated polysaccharides and complex cell-wall matrix	LAB, agarase- or carrageenase-producing bacteria, and yeast/LAB co-fermentation	Acidification, proteolysis, flavor improvement, antioxidant changes, and partial polysaccharide degradation	Costa et al. (2022) and Yang et al. (2022)
Green macroalgae, e.g., *Ulva* and *Codium*	Cellulose, ulvan, soluble sugars, proteins, minerals, and phenolic compounds	Cross-linked cellulose–ulvan matrix and limited accessibility of intracellular nutrients	Ulvan lyase or carbohydrase pretreatment followed by LAB or yeast fermentation	Increased release of soluble sugars, improved substrate accessibility, and changes in nutritional composition	Costa et al. (2022) and Ferreira et al. (2022)

### 5.2 Lactic acid fermentation (LAF)

Algae are rich in a diverse array of nutrients that promote microbial proliferation, making them suitable substrates for fermentation with inoculants such as LAB and yeasts. This fermentation process yields several valuable by products for the food industry, including acetoin, 2,3-butanediol, higher alcohols, glycerol, diacetyl, esters, and succinic acid (Babich et al., 2024). Fermentation of algal biomass can generate organic acids, alcohols, volatile compounds, peptides, and other value-added metabolites. However, the production of these compounds is strongly dependent on the algal species, pretreatment method, microbial strain, pH, temperature, and fermentation time. For example, acid–thermal hydrolysis followed by LAF was applied to *Ulva* sp., *Gracilaria* sp., and *Sargassum cristaefolium* macroalgae. Using sulfuric acid concentrations below 5% for hydrolysis, *Gracilaria* sp. produced a maximum reducing-sugar recovery of 0.39 g/g biomass and a lactic acid yield of 0.94 g/g. The corresponding lactic acid yields were 0.85 g/g for *Ulva* sp. and 0.81 g/g for *S. cristaefolium* (Nagarajan et al., 2022). In another study, fermentation of *Alaria esculenta* by a lactic acid bacterial consortium resulted in a pH decrease to approximately 3.8 after 7 days. The consortium utilized glucose and laminari-oligosaccharides and produced lactic acid, whereas mannitol fermentation generated lactic acid, ethanol, and succinate (Allahgholi et al., 2023). These results demonstrate that quantitative evaluation of fermentation performance should include substrate conversion, product concentration, yield, productivity, pH reduction, viable-cell counts, and sensory changes rather than only reporting the presence of fermentation-derived metabolites.

Lactic acid, produced through the fermentation of carbohydrates derived from algae, can inhibit the growth of spoilage and pathogenic microorganisms, thus extending the shelf life of food products. Beyond its function in food preservation, lactic acid holds substantial potential in pharmaceuticals, textiles, cosmetics, and packaging, especially as a precursor for polylactic acid, a biodegradable substitute for petroleum - based polymers (Lin et al., 2020). Algae rich in carbohydrates can be effectively converted into lactic acid via fermentation. Additionally, co-fermentation strategies have been devised to enhance the production of organic acids. For instance, when the brown algae *Saccharina japonica* is co-fermented with rice straw, it promotes butyric acid production by providing carbon sources to compensate for the inefficient utilization of mannitol by *Clostridium tyrobutyricum* (Oh et al., 2019).

Lactic acid fermentation of algae biomass is utilized not only for lactic acid production but also for the development of functional beverages and foods through the generation of organic acids and phenolic compounds. Moreover, LAB-based ensiling of algae can enhance storage stability while improving the bioactivity of the final product. A variety of photosynthetic organisms, including microalgae, macroalgae, and cyanobacteria, have been employed in LAF processes. Commonly used microorganisms include *Lactobacillus*, *Lacticaseibacillus*, *Lactococcus*, *Tetragenococcus*, *Bacillus*, *Candida albicans*, and *Weissella*. LAF can be carried out under both liquid and solid–state fermentation conditions.

### 5.3 Alcoholic fermentation

Alcoholic fermentation represents a bioconversion mechanism wherein microorganisms, chiefly *Saccharomyces cerevisiae*, metabolize sugars to generate ethanol and carbon dioxide, along with a variety of by products such as ethyl acetate, 2,3-butanediol, higher alcohols, glycerol, diacetyl, esters, and succinic acid (Koller, 2023). Yeasts can contribute to algal fermentation through sugar conversion, aroma formation, protein hydrolysis, and biotransformation of undesirable odor compounds. Compared with lactic acid bacteria, yeasts are particularly valuable when the primary objective is to improve the sensory properties of algae-based foods. *Saccharomyces cerevisiae* has been used to modify the volatile profile of brown and red seaweeds. During fermentation of Saccharina japonica seedlings, the number and composition of volatile compounds changed substantially, including alcohols, aldehydes, ketones, alkanes, alkenes, and esters. Several compounds associated with fishy or marine odors, such as 1-octen-3-ol and hexanal, were reduced after fermentation (Lin et al., 2020). Similarly, fermentation of *Bangia fusco-purpurea* with *S. cerevisiae* reduced or eliminated several odor-active compounds, including 1-octen-3-ol, hexanal, nonenal, 2-pentylfuran, and unsaturated aldehydes. These changes were associated with the formation of less undesirable alcohols and other volatile compounds (Gong et al., 2024).

Yeast fermentation may also affect the biological properties of algal products. Fermentation of seaweed extracts using *S. cerevisiae* and *Lactiplantibacillus plantarum* increased bile-acid-binding capacity and pancreatic-lipase-inhibitory activity *in vitro*, and the fermented extracts showed beneficial effects in a hyperlipidemic mouse model (Xu et al., 2022). Nevertheless, these biological effects should not be generalized to all yeast-fermented algae because they depend on the strain, algal substrate, fermentation conditions, and post-fermentation processing.

The use of yeasts for algal fermentation also has limitations. Conventional *S. cerevisiae* strains efficiently consume glucose and other readily fermentable sugars but generally show limited capacity to utilize alginate and other unusual brown-algal carbohydrates. Therefore, enzymatic pretreatment, adaptive evolution, metabolic engineering, or co-culture with polysaccharide-degrading bacteria may be required when brown-algal polysaccharides are the main carbon source (Du et al., 2021). Conventional algae processing techniques, such as drying and roasting, frequently fail to effectively disrupt cell wall structures, leading to the restricted bioavailability of intracellular bioactive compounds. Fermentation with yeasts can enhance the sensory attributes and digestibility of algal products while promoting the release of bioactive metabolites, as evidenced in the fermentation of nori (Sarıtaş et al., 2024). This process also improves the nutritional composition of algae, facilitating their integration into diverse food formulations.

Apart from ethanol, algae fermentation can produce a range of high-value products through microbial bioconversion, including organic acids, enzymes, proteins, and pigments. Therefore, algal biomass holds substantial potential as a sustainable substrate for alcoholic fermentation, with diverse output prospects and the ability to supplement or partially substitute traditional terrestrial crop resources.

### 5.4 Mixed fermentation

Mixed-culture fermentation technology, which entails the integration of diverse microorganisms or substrates, represents a promising approach for overcoming the limitations of single-strain processes. This methodology promotes synergistic interactions that augment product yield and broaden the spectrum of utilizable substrates. Algae feature robust cell walls that frequently require physical or chemical pretreatment to disrupt their structural integrity, thereby enabling microorganisms to access intracellular nutrients for fermentation (Tabacof et al., 2024). This limitation can be biologically alleviated in mixed fermentation systems. Specifically, filamentous fungi secrete copious extracellular enzymes that degrade algal cell walls. This enzymatic hydrolysis facilitates the subsequent inoculation of LAB for fermentation. The lactic acid produced not only contributes to the product composition but also establishes a selective environment by reducing the pH, which inhibits the growth of undesirable microorganisms. This stabilized system provides favorable conditions for subsequent yeast inoculation. During alcoholic fermentation, ethanol produced by yeast can react with organic acids originating from LAB metabolism to generate various esters. Concurrently, yeast metabolism yields a variety of flavor-active compounds, including higher alcohols, aldehydes, and ketones, which collectively enhance the complexity and sensory richness of the final fermented product (Liszkowska and Berlowska, 2021).

### 5.5 Comparison of fermentative microorganisms

Different microorganisms provide distinct advantages during the fermentation of edible algae. LAB are generally preferred for food applications because of their established history of safe use, acidification capacity, and ability to produce organic acids and antimicrobial metabolites. They are particularly suitable for improving microbiological stability and reducing undesirable marine notes, although their ability to degrade complex algal polysaccharides is often limited. Bacillus species generally display stronger proteolytic and polysaccharide-degrading activities than many lactic acid bacteria. They may therefore be useful for improving protein digestibility and releasing bioactive peptides. However, strain-level differences are substantial, and the possible formation of biogenic amines, ammonia, or undesirable volatile compounds must be evaluated. Yeasts are valuable for flavor development because they produce ethanol, esters, higher alcohols, and other volatile compounds. Their application is suitable when the main objective is to reduce algal off-flavors or generate fermented beverage and seasoning products. Nevertheless, yeasts may not efficiently utilize highly sulfated or structurally complex algal polysaccharides. Filamentous fungi possess extensive extracellular enzymatic systems and may promote more pronounced degradation of algal biomass. Their use can improve the release of soluble nutrients and bioactive compounds, but it also presents greater safety and process-control challenges, particularly with respect to strain authentication and mycotoxin production. Mixed cultures may combine the advantages of different microorganisms, but they require more complex optimization and process monitoring (Table 3).

**Table 3:** Comparison of microorganisms used for edible-algae fermentation.

Microorganism	Main enzymes/pathways	Major effects on edible algae	Typical metabolites	Advantages	Limitations	References
*Lactic acid bacteria (LAB)*	Glycosidases and peptidases; homo- or heterofermentative lactic-acid pathways; strain-dependent utilization of mannitol and laminarin-derived oligosaccharides	Acidification, partial hydrolysis of proteins and carbohydrates, improvement of microbiological stability, and reduction of undesirable marine odors	Lactic acid, acetic acid, CO₂, bacteriocin-like compounds, and aroma-active metabolites	Many LAB strains have an established history of food use and are suitable for fermented functional foods	Their capacity to degrade alginate, carrageenan, and other highly complex sulfated polysaccharides is generally strain-dependent and may be limited	Healy et al. (2023), Lin et al. (2020), and Yang et al. (2022)
*Bacillus* spp.	Proteases, alginate lyases, cellulases, amylases, and other hydrolytic enzymes	Disruption of algal biomass, protein hydrolysis, release of peptides and soluble carbohydrates, and depolymerization of alginate	Peptides, amino acids, organic acids, ammonia, and volatile compounds	Strong extracellular hydrolytic capacity and potential for alginate degradation	Strain-level differences are substantial; proteolysis may generate bitterness, ammonia, or biogenic amines, so strain selection and safety evaluation are required	Sun et al. (2020), Sun et al. (2023), and Wang et al. (2016)
*Saccharomyces* spp.	Glycolytic and alcoholic-fermentation pathways; carbohydrate utilization and aroma-biotransformation reactions	Conversion of available sugars, reduction of fishy or green odors, and development of fermented beverage or seasoning characteristics	Ethanol, CO₂, esters, higher alcohols, aldehydes, and other volatile compounds	Well-established food application and strong potential for flavor development and deodorization	Yeasts may not efficiently utilize highly sulfated or structurally complex algal polysaccharides without pretreatment or co-culture	Du et al. (2021), Gong et al. (2024), and Xu et al. (2022)
*Acetic acid bacteria*	Membrane-bound alcohol dehydrogenase and aldehyde dehydrogenase; oxidative conversion of ethanol to acetic acid	Acidification, oxidation of yeast-derived ethanol, and modification of odor-active compounds	Acetic acid, aldehydes, esters, and other volatile compounds	Useful for acidification and flavor modulation, particularly in sequential or mixed fermentations	Requires sufficient oxygen; excessive acetic-acid formation may negatively affect taste and product stability	Du et al. (2021) and Li et al. (2023)
*Filamentous fungi*	Cellulases, hemicellulases, proteases, glycosidases, oxidoreductases, and other carbohydrate-active enzymes	Extensive deconstruction of algal biomass, release of soluble nutrients, and production of extracellular enzymes	Organic acids, peptides, amino acids, enzymes, and volatile compounds	Broad enzymatic repertoire and potential for solid-state fermentation	Strict strain authentication and monitoring of unwanted secondary metabolites, including mycotoxins, are necessary; process control can be more demanding	Ferreira et al. (2022) and Le Strat et al. (2022)
*Mixed cultures*	Complementary hydrolytic, acidogenic, alcoholic, and oxidative pathways; microbial cross-feeding and succession	Enhanced substrate conversion, improved flavor complexity, and simultaneous modification of nutritional and sensory properties	Organic acids, ethanol, esters, peptides, bacteriocin-like compounds, and other bioactive metabolites	Functional complementarity and potentially better performance than monocultures	More difficult to control, standardize, and reproduce because of microbial competition and changes in community composition	Healy et al. (2023) and Yue et al. (2021)

Overall, microorganism selection should be based on the target alga, the desired product, the dominant cell-wall polymers, the intended metabolic products, and the required safety profile. A microorganism with high hydrolytic activity is not necessarily the best choice for food production if it produces undesirable flavors or toxic metabolites.

## 6 Challenges, strategies, and prospects

Although algae are promising sustainable food resources, their wider application remains constrained by sensory acceptance, safety risks, insufficient characterization of functional components, and immature processing technologies. These limitations should be addressed through integrated strategies involving strain selection, process optimization, safety regulation, and product-oriented development.

### 6.1 Sensory acceptance and food safety

Consumer acceptance remains a major barrier to the commercialization of algal foods because marine or fish-like odors, mainly associated with volatile sulfur compounds and lipid-oxidation aldehydes, may reduce consumer preference (Blikra et al., 2021). Fermentation can improve sensory quality by reducing undesirable volatiles and generating favorable acids, esters, and aroma compounds; however, uncontrolled fermentation may also produce undesirable metabolites. Therefore, sensory optimization should be integrated into fermentation-process design.

Food safety risks may arise from heavy metals, excessive iodine, arsenic species, and microbial contamination accumulated during cultivation and processing (Banach et al., 2020). During fermentation, decarboxylase-positive lactic acid bacteria may convert algal free amino acids into biogenic amines such as histamine and tyramine, whereas uncontrolled mold growth or poorly characterized fungi used in solid-state fermentation may result in mycotoxin production. Therefore, food-grade microorganisms should be authenticated and screened for biogenic amine- or mycotoxin-producing potential, while pH, temperature, water activity, fermentation time, and microbial purity should be carefully controlled (Ma et al., 2021; Shen et al., 2024). Fermentation should not be assumed to remove heavy metals, iodine, or other chemical contaminants; species-specific monitoring and final-product testing remain necessary for ensuring product safety and regulatory compliance (European Food Safety et al., 2023).

### 6.2 Knowledge gaps in functional composition and bioavailability

Despite their high nutritional value, the digestibility and bioavailability of algal proteins and other bioactive compounds remain insufficiently understood. Current protein extraction and purification methods are often inefficient or poorly adapted to algal matrices. Moreover, many algal polysaccharides, including carrageenan and agarose, require mild and selective extraction conditions to preserve their native structures and functions.

Another unresolved issue is the complexity of algal secondary metabolites. Their biological effects may depend not only on individual compounds but also on synergistic interactions among multiple components. Similar structure-dependent interactions have been observed for humic substances during biomass conversion processes (Wang X. et al., 2021), suggesting that comparable mechanisms may exist in algal systems. Clarifying these interactions is necessary for the precise use of algal ingredients in functional foods, pharmaceuticals, and other high-value applications.

### 6.3 Processing bottlenecks and low overall utilization

The industrial application of algae remains constrained by limited biomass productivity, high production costs, and inefficient downstream processing. Compared with major terrestrial crops such as wheat, current large-scale microalgal production is still substantially lower, which limits the competitiveness of algae as bulk food or feed ingredients (Vigani et al., 2015). Therefore, algae are currently more suitable as high-value ingredients, nutritional enhancers, or sources of specific bioactive compounds than as direct replacements for conventional staple crops.

Downstream processing is another major technical and economic bottleneck. Conventional extraction methods often require long processing times, high energy inputs, and environmentally undesirable solvents, while providing limited selectivity and recovery efficiency (Cardoso et al., 2025). In addition, algal hydrolysates may contain fermentation inhibitors that require detoxification before microbial conversion, increasing process complexity and cost (Tabacof et al., 2024). The limited availability of efficient and specific enzymes for degrading complex algal polysaccharides further restricts the complete utilization of algal biomass (Rinaldi et al., 2020). These limitations highlight the need for integrated biorefinery strategies that recover high-value compounds while converting residual biomass into food or feed ingredients, bioenergy, and other co-products.

The scale-up of fermentative algal valorization is further complicated by variations in species, cultivation conditions, season, harvest time, and post-harvest handling. Such variability can alter the protein, carbohydrate, lipid, ash, salt, and moisture contents of the biomass and consequently affect fermentation performance. Pretreatment may be necessary to disrupt algal cell walls and improve substrate accessibility; however, excessive chemical or thermal treatment may increase energy consumption, degrade valuable compounds, and generate fermentation inhibitors. At larger scales, fermentation reactors must also address mixing, oxygen and heat transfer, pH control, viscosity, and contamination. Subsequent separation, concentration, drying, and stabilization steps may further increase production costs. Consequently, the feasibility of fermented algal ingredients should be assessed using pilot-scale data, realistic biomass and product yields, energy and water requirements, downstream recovery efficiencies, and market prices. Techno-economic analysis and life-cycle assessment should be integrated into process development to determine whether fermented algal products can compete with conventional food ingredients in terms of cost, environmental performance, functionality, and safety.

### 6.4 Improvements and prospects to algae in food applications

Fermentation should be integrated into a cascading algal biorefinery rather than treated as an isolated processing step. High-value compounds, including proteins, pigments, lipids, polyphenols, and specialty polysaccharides, can first be recovered, while the remaining carbohydrate- or protein-rich fractions can be fermented to produce organic acids, alcohols, peptides, enzymes, and aroma compounds. Residual solids may subsequently be directed toward food or feed ingredients, anaerobic digestion, soil amendments, biochar, or other non-food applications, provided that their safety and contaminant profiles are acceptable. Such multi-product processing can improve whole-biomass utilization, reduce waste generation, and distribute production costs across several product streams. However, the optimal process configuration depends on algal species, biochemical composition, product requirements, regulatory constraints, and the safety of each biomass fraction.

Future development should focus on three closely connected priorities. First, a broader range of algal species should undergo systematic nutritional, toxicological, and microbiological safety assessments to support regulatory approval and, where appropriate, GRAS recognition. This would expand the number of species available for food applications and facilitate their integration into biorefinery systems. Second, strain selection and improvement should be strengthened. Unlike conventional crops, most edible algae have undergone limited domestication. Promising strains could therefore be selected and improved through classical breeding, mutagenesis, adaptive evolution, or genetic engineering, where legally and ethically acceptable. Model organisms such as *Chlamydomonas reinhardtii* may provide useful platforms for identifying traits related to biomass productivity, nutrient composition, stress tolerance, and metabolite production (Fields et al., 2020).

Third, cultivation and processing conditions should be optimized according to strain-specific metabolic characteristics. Biomass productivity and composition are strongly influenced by medium formulation, light intensity, temperature, photoperiod, cultivation mode, and bioreactor configuration (Feng et al., 2014; Guccione et al., 2014). Statistical methods, including response-surface methodology and Box–Behnken designs, can facilitate the optimization of cultivation and fermentation parameters (Kanaga et al., 2016). For food applications, improvements in nutritional composition should be accompanied by sensory optimization. High-throughput screening, electronic-tongue analysis, human sensory evaluation, and targeted processing may help reduce algal off-flavors and improve palatability. Genetic engineering and controlled processing reactions, including Maillard-based flavor development, may provide additional strategies for improving the sensory quality of algal foods (Torres-Tiji et al., 2020).

Overall, the future value of algae in sustainable food systems will depend on the coordinated development of safe cultivation, strain improvement, sensory optimization, efficient downstream processing, and economically viable whole-biomass utilization. Rather than serving as direct replacements for staple crops, algae may be more realistically developed as high-value functional ingredients within diversified, multi-product, and sustainable algal biorefinery systems.

## 7 Conclusion

Edible algae represent a sustainable and nutritionally valuable feedstock for future food systems, providing proteins, polyunsaturated fatty acids, pigments, minerals, and functional polysaccharides; however, their broader utilization is limited by species-dependent biomass composition, recalcitrant cell-wall structures, poor nutrient bioaccessibility, marine off-flavors, potential contaminants, and the high costs of harvesting, pretreatment, fermentation, and downstream processing. Fermentation offers an effective biological upgrading strategy by promoting cell-wall deconstruction, improving nutrient digestibility and bioavailability, releasing bioactive peptides and oligosaccharides, modulating flavor-active compounds, and converting residual biomass into value-added products. Future research should therefore establish standardized, species-specific fermentation protocols that optimize pretreatment, substrate loading, microbial strain selection, inoculum size, pH, temperature, oxygen transfer, agitation, and fermentation time. Strain improvement and metabolic engineering should be integrated with omics-based approaches to elucidate strain–substrate interactions and enhance the targeted production of organic acids, peptides, enzymes, aroma compounds, and other functional metabolites. Pilot-scale studies should further assess biomass-supply consistency, process reproducibility, contaminant control, mass and heat transfer, downstream recovery, product stability, shelf life, and consumer acceptance, while life-cycle assessment and techno-economic analysis should determine the competitiveness of fermented algal ingredients relative to conventional food ingredients. Ultimately, the integration of precision pretreatment, robust fermentation control, matrix-specific formulation, standardized safety management, and cascading biorefinery design will be essential for translating algal fermentation from laboratory research into a safe, reproducible, scalable, and commercially viable platform for multifunctional food ingredients.
